# Social Behavior of Pet Dogs Is Associated with Peripheral OXTR Methylation

**DOI:** 10.3389/fpsyg.2017.00549

**Published:** 2017-04-10

**Authors:** Giulia Cimarelli, Zsófia Virányi, Borbála Turcsán, Zsolt Rónai, Mária Sasvári-Székely, Zsófia Bánlaki

**Affiliations:** ^1^Clever Dog Lab, Comparative Cognition, Messerli Research Institute, University of Veterinary Medicine Vienna, Medical University of Vienna, University of ViennaVienna, Austria; ^2^Wolf Science CenterErnstbrunn, Austria; ^3^Department of Cognitive Biology, University of ViennaVienna, Austria; ^4^Institute of Cognitive Neuroscience and Psychology, Research Centre for Natural Sciences, Hungarian Academy of SciencesBudapest, Hungary; ^5^Department of Medical Chemistry, Molecular Biology and Pathobiochemistry, Semmelweis UniversityBudapest, Hungary

**Keywords:** dog, DNA methylation, epigenetics, social behavior, oxytocin receptor gene, ownership style, oxytocin

## Abstract

Oxytocin is a key modulator of emotional processing and social cognitive function. In line with this, polymorphisms of genes involved in oxytocin signaling, like the oxytocin receptor (*OXTR*) gene, are known to influence social behavior in various species. However, to date, no study has investigated environmental factors possibly influencing the epigenetic variation of the *OXTR* gene and its behavioral effects in dogs. Pet dogs form individualized and strong relationships with their owners who are central figures in the social environment of their dogs and therefore might influence the methylation levels of their *OXTR* gene. Here we set out to investigate whether DNA methylation within the *OXTR* promoter region of pet dogs is linked to their owner’s interaction style and to the social behavior of the dogs. To be able to do so, we collected buccal epithelial cells and, in Study 1, we used pyrosequencing techniques to look for differentially methylated CpG sites in the canine *OXTR* promoter region on a heterogeneous sample of dogs and wolves of different ages and keeping conditions. Four identified sites (at positions -727, -751, -1371, and -1383 from transcription start site) showing more than 10% methylation variation were then, in Study 2, measured in triplicate in 217 pet Border Collies previously tested for reactions to an adverse social situation (i.e., approach by a threatening human) and with available data on their owners’ interaction styles. We found that CpG methylation was significantly associated with the behavior of the dogs, in particular with the likelihood that dogs would hide behind their owner or remain passive when approached by a threatening human. On the other hand, CpG methylation was not related to the owners’ behavior but to dog sex (at position -1371). Our findings underpin the complex relationship between epigenetics and behavior and highlight the importance of including epigenetic methods in the analysis of dog behavioral development. Further research is needed to investigate which environmental factors influence the epigenetic variation of the *OXTR* gene.

## Introduction

Social interactions are central to the life of all social species, and genetic variation across individuals has been associated with mechanisms regulating these interactions. In particular, associations have been found between the genetic variation of different genes involved in the oxytocinergic system and a variety of social phenotypes in different mammalian species, e.g., mice (see Caldwell et al., 2016 for a review), primates (Staes et al., 2014), cats (Arahori et al., 2015), humans (see Ebstein et al., 2012 for a review), and dogs (Kis et al., 2014). For example, polymorphisms in the oxytocin receptor (*OXTR*) gene were associated with dogs’ proximity seeking with the owner (Kis et al., 2014), rough temperament in cats (Arahori et al., 2015), and sociability in humans (Li et al., 2015). Furthermore, oxytocin has been associated with social fear (Kirsch et al., 2005), aggression toward unfamiliar individuals (Stallen et al., 2012) and social anxiety (Grillon et al., 2013) in humans, and friendliness toward a threatening human in dogs (Hernádi et al., 2015). In particular, Hernádi et al. (2015) showed that dogs, after intranasal oxytocin administration, showed less friendliness toward the owner approaching them in a threatening way (in the so-called Threatening Approach test, originally developed and validated by Vas et al. (2005, 2008) and looked more at their owners standing behind them than a control group of dogs who received a placebo. These results, taken together with the associations between *OXTR* polymorphisms and dog friendliness and proximity seeking toward the owner found by Kis et al. (2014) during the same test, highlight a potential dual role of the oxytocinergic system: regulating a dog’s behavioral response toward a social threat and expressing the relationship between the dog and the owner present in such a situation.

The relationship dogs build with their owners (at least in western societies) has been defined as analogous to the infant-mother attachment bond (Topál et al., 1998), and it has been shown that the presence of the owner influences the coping strategy of a dog exposed to such social threats (Horváth et al., 2007) and attachment-related behaviors like proximity seeking (Gácsi et al., 2013). This latter behavior has been interpreted as dogs seeing their owners as a “safe haven,” a concept introduced by Bowlby (1969) in the frame of the attachment theory. The safe haven effect is activated by distress and fear, when a child (or a dog) seeks for proximity to the caregiver in order to find protection and safety. However, it is important to notice that not all dogs seek proximity in the same way, suggesting that individual differences might play a role in shaping the relationship between a dog and its owner. In fact, it has been shown that the reactions of dogs during the Threatening Approach test are strictly associated with the interaction styles of their owners (Cimarelli et al., 2016), supporting the idea that owners can indeed serve as a safe haven for their dogs, but only if they show specific behavioral characteristics. Specifically, only if previous experiences provided the dog with the information that the caregiver was present and responsive.

Similarly, in human infants, the caregivers’ parenting style strongly influences the children’s attachment styles (that is, their behavioral reaction to separation from and reunion with the caregiver), and it has been proposed that epigenetic modifications of the genome are the biological mechanisms that mediate this link between caregiver and child behavior (Champagne and Curley, 2009). In fact, epigenetic modifications of the DNA, that affect gene expression but do not alter the primary sequence itself, are known to be influenced by various biological and environmental factors (Powledge, 2011; Tammen et al., 2013). One of their best known mechanisms is DNA methylation that in mammals occurs predominantly on cytosine residues that are followed by guanine (CpG sites). Although DNA methylation can exert opposite effects on transcription efficiency depending on the genomic context and extent of methylation, basically it represses gene expression. This is especially true for promoter regions where DNA methylation is considered as a major factor influencing gene expression (e.g., in tissue-specific transcriptional inactivation, Goldberg et al., 2007; Portela and Esteller, 2010). Studies in rodents show that the social environment in which individuals grow up, and as such, also caregiving quality, has a high impact on DNA methylation (Weaver et al., 2004; McGowan et al., 2011). In child development it has been suggested that epigenetic modifications of specific genes caused by environmental factors result in changes in emotion regulation and, in turn, in behavior (van Ijzendoorn et al., 2011). For example, it has been shown that methylation levels in the hippocampus of suicidal victims who had experienced abuse was higher than in individuals who committed suicide but had no history of abuse (McGowan et al., 2009). Also, children with mothers reporting a warmer and more affectionate caregiving style had lower methylation levels in the glucocorticoid receptor gene (Bick et al., 2012). Regarding the role of the oxytocinergic system in the epigenetic modification of social behavior, it seems that this system is influenced by early experience (e.g., Unternaehrer et al., 2015), and it has been suggested that methylation of the *OXTR* gene mediates the effect of parental care on psychosocial development in humans (MacDonald, 2012; MacDonald and Feifel, 2013; Shalev and Ebstein, 2013; Feldman, 2015) and in rodents (Zhang et al., 2010; Bales and Perkeybile, 2012). In humans, a possible role of *OXTR* methylation in behavioral neuroscience is also underpinned by functional gene expression studies (Kusui et al., 2001) and by observations on the relationship between *OXTR* methylation and psychosocial traits (Kumsta et al., 2013). In particular, *OXTR* methylation has been linked to autism (Gregory et al., 2009), social perception (Jack et al., 2012), callous-unemotional traits (Dadds et al., 2014), anxiety/depression (Chagnon et al., 2015) as well as anger and fear perception (Puglia et al., 2015). *OXTR* methylation levels have also been shown to change dynamically upon acute psychosocial stress (Unternaehrer et al., 2012).

Here we suggest that epigenetic mechanisms are also likely to play a role in mediating the effects of the owner behavior on the social behavior of pet dogs. We have shown that dog owners’ interaction styles vary along three components that are analogous to components of human parenting styles and that they are associated with how dogs cope with a socially stressful situation (Cimarelli et al., 2016). We hypothesize that methylation of the *OXTR* gene may play a role in mediating such a link between owner and dog behavior. Investigating possible causes and effects of differential methylation patterns in the dog *OXTR* gene is not only relevant for the field of anthrozoology or canine behavior but could represent a valid model of human caregiving behavior and its effects on the social behavior of cared individuals (either children or dogs). In fact, the vast majority of animal studies have so far focused mainly on laboratory rodents that live in environments not comparable to that of humans. Also, rodent maternal behavior, albeit possibly analogous the human parenting, still has a rather different biological function. It has been pointed out that it is difficult to retrieve useful information from comparing the “reproductive and parenting strategies of humans and other species” (Galbally et al., 2015, p. 2). In contrast, in pet dogs we can directly investigate the effects of human caregiving on dog social behavior. Furthermore, dogs share their environment with humans, not only in terms of habitat and nutrition but also of communication and social interactions (Hare and Tomasello, 2005; Tomasello and Kaminski, 2009; Miklósi and Topál, 2013). Therefore, pet dogs might provide a more relevant animal model than laboratory rodents for studying associations between epigenetic variables and behavior. Finally, this species is also genetically uniquely suited to investigate the genetic background and gene-related associations of various behavior traits (Hejjas et al., 2007). Purebred dogs show a genetic diversity that is intermediate between two extremes represented by the genetically highly variable humans and the genetically nearly homogeneous laboratory animal strains. This intermediate genetic diversity can facilitate the identification of genetic factors underlying phenotypic variation (Ostrander, 2005; Boyko, 2011; Parker, 2012). Despite of these advantages, to date few studies have investigated epigenetic variation in the domestic dog (Maeda et al., 2014; Tomiyasu et al., 2014; Berglund et al., 2015; Montrose et al., 2015; Yamaya et al., 2015) and none of them focused on associations between the *OXTR* gene and behavioral phenotype.

Here, in Study 1, we explored differentially methylated CpG sites within the canine *OXTR* promoter region in a heterogeneous sample of dogs and wolves living in different social environments in order to describe the epigenetic variation of the canine *OXTR* promoter. Then, in Study 2, considering the hypothesis that different owner interaction styles might have an effect on dog behavior through *OXTR* methylation, first we investigated possible relationships between methylation levels on specific regions of the *OXTR* promoter and the dogs’ behavioral reactions to an unpleasant social situation (including experimenter-directed behaviors and owner-directed behaviors, e.g., proximity seeking) in a large sample of pet Border Collies. Second, in the same dogs we investigated whether dog-directed interaction styles of the owners are associated with DNA methylation levels of *OXTR* gene promoter of the dogs.

## Materials and Methods

### Ethics Statement

No special permission for non-invasive sample taking and socio-cognitive testing of animals is required either in Austria (Tierversuchsgesetz 2012 – TVG 2012) or in Hungary (Act XXVIII of 1998 on the protection and welfare of animals). In accordance with GPS guidelines and national legislation, the experimental procedures of Study 2 were approved by the Ethical Committee for the use of animals in experiments at the University of Veterinary Medicine Vienna (Ref: 09/10/97/2012 and 10/10/97/2012). Owners of the pet dogs participated in the study on a voluntary basis and gave their consent to the genetic analyses as well as the behavioral testing of their dogs.

### Sample Collection and DNA Isolation

DNA samples were collected from the inner cheek of the animals using cotton-tipped swabs. Genomic DNA was isolated by a traditional, salting-out procedure as described earlier (Boor et al., 2002). Briefly, collection swabs were incubated overnight at 56°C in 450 μl cell lysis buffer (0.2 g/l Proteinase K, 0.1 M NaCl, 0.5% SDS, 0.01 M Tris buffer pH = 8.0), RNase treated at room temperature, protein precipitated with saturated NaCl (6 M) and centrifuged. DNA was obtained by precipitating the supernatants with isopropanol. Following ethanol purification, pellets were resuspended in 50 μl of Tris-EDTA (0.01 M Tris, 0.001 M EDTA, pH = 8.0) and stored at -20°C prior to bisulfite conversion.

### DNA Methylation Analysis

Two hundred nanograms genomic DNA quantified by a NanoDrop ND-1000 Spectrophotometer (Thermo Scientific, Wilmington, DE, USA) was bisulfite converted using the EZ DNA Methylation-Gold^TM^ Kit (Zymo Research, Irvine, CA, USA) according to the manufacturer’s protocol. Bisulfite converted DNA was kept at -80°C until further used. Primers were designed to bisulfite converted regions of an approximately 1000 base pairs (bp) long CpG island shore stretch at the canine *OXTR* promoter/ 5′ untranslated region (UTR) by the PyroMark Q24 Software (Qiagen NV, Venlo, Netherlands). CpG island localization was determined by an in-house MS-DOS application using the traditional definition of a CpG island as *a* ≥ 200 bp long region with a GC percentage >50% and an observed-to-expected (O/E) CpG ratio greater than 60%. The *OXTR* promoter was located according to genome assembly CanFam 3.1 (GCA_000002285.2) and CpG sites investigated were numbered according to transcription start site (+1) of transcript variant NM_001198659.1 (ENSCAFT00000008950.3; genomic coordinate Chr20:9358932) (Aken et al., 2016). For polymerase chain reaction (PCR) amplification, the 25 μl reaction mixture contained 0.625 units EpiMark Hot Start Taq DNA Polymerase (New England Biolabs, Ipswich, MA, USA), 1x EpiMark Hot Start Taq Reaction Buffer (New England Biolabs, Ipswich, MA, USA), 0.2 mM deoxynucleotide trisphosphate (dNTP), 10 μM of an unmodified forward primer and a biotin-labeled reverse primer (for sequences see **Table 1**) and about 15–20 ng bisulfite converted DNA template. All samples were amplified in triplicate on the same PCR machine (Bio-Rad T100^TM^). Cycling conditions were as follows: Step 1: (95°C/ 1 min)/1 cycle; Step 2: (95°C/30 s, 58°C/1 min, 68°C/45 s)/45 cycles; Step 3: (68°C/5 min)/1 cycle; Step 4: 8°C hold. Successful PCR amplification of a single fragment was verified using agarose gel electrophoresis for each sample and replicate. Pyrosequencing was performed on a PyroMark Q24 platform using sequencing primers indicated in **Table 1** with PyroMark Gold Q24 Reagents (Qiagen NV, Venlo, Netherlands). Totally methylated and absolutely unmethylated control DNA were obtained by SSSI methyltransferase treatment (New England Biolabs, Ipswich, MA, USA) and whole genome amplification (REPLI-g Mini Kit, Qiagen NV, Venlo, Netherlands), respectively, according to the manufacturers’ protocols. Measurements reported as unreliable by the PyroMark software were removed from the database. Epigenotypes reported are an average of triplicate measurements (outliers, i.e., values deviating more than 3% were removed).

**Table 1:** Primers used for the exploration of differentially methylated CpG sites in the canine *OXTR* promoter.

Primer Name	Sequence	Genomic coordinates (Chromosome 20)	Type	Quality score
P1_F	5′ TGA TGT AAT TTT TAA GGG TAA GAA AAA GAT A 3′	9357389 : 9357419	Amp	–
P1_R	5′ TTT AAA TAC ATT CTT CCT CCT AAC ATT TCC TTT C 3′	9357608 : 9357641	Amp	–
P1_S1	5′ AAT TTT TAA TTT TTT TTA ATG TTG T 3′	9357419 : 9357442	Seq	74
P1_S2	5′ TTA ATT AGA ATT TTG GGA TT 3′	9357476 : 9357495	Seq	76
P1_S3	5′ GGT ATA GGG TTG TAA TTG 3′	9357530 : 9357547	Seq	79
P2_F1	5′ AGG GTG ATG AAG TTG TAA AAG T 3′	9358139 : 9358160	Amp	–
P2_F2	5′ AGG GAA AGA TTT TAA GAA AAG ATA AGA AAG 3′	9357913 : 9357938	Amp	–
P2_R	5′ ACA TTT CAT CTT CCT TTA ACA TCA TAT A 3′	9357788 : 9357815	Amp	–
P2_S1	5′ ATG AAG TTG TAA AAGTAT TTA ATT G 3′	9358130 : 9358154	Seq	71^∗^
P2_S2	5′ TAA GTA AAT GTT TGT TTT GGA GTA 3′	9358026 : 9358049	Seq	68^∗^
P2_S3	5′ AAT TTA TTT TTA TTT TAA AGT GAT T 3′	9357875 : 9357899	Seq	80^#^
P3_F	5′ GG TTT TTG GAT GGG GAT AGG A 3′	9358485 : 9358505	Amp	–
P3_R	5′ ACT TCA TCA CCC TCT TCT CA 3′	9358148 : 9358167	Amp	–
P3_S1	5′ TTT TTG GAT GGG GAT AGG 3′	9358486 : 9358503	Seq	68
P3_S2	5′ GGT AGG AGG TAA AAA AAA G 3′	9358450 : 9358468	Seq	68
P3_S3	5′ GTT GGG GAG AGT TTT TTT GTA GT 3′	9358416 : 9358438	Seq	69
P3_S4	5′ GTA TAG TTT TAA GGG GTT ATT GGG 3′	9358378 : 9358401	Seq	72
P3_S5	5′ ATT TTT AGA TTA GGG TTA GTT TGG A 3′	9358330 : 9358354	Seq	72
P3_S6	5′ AAT TAG TAG TTT TAT TTT ATT TAA G 3′	9358288 : 9358312	Seq	69
P3_S7	5′ GGT TTT TTT TTT TTT TGG TTT AGA A 3′	9358217 : 9358241	Seq	63


*Genomic coordinates are according to CanFam3.1 (GCA_000002285.2). Quality scores (<40: poor; 40–59: low; 60–87: medium; > 88: high quality) are assigned as by the PyroMark Assay Design Software and refer to primer sets (including forward, reverse and sequencing). Amp: amplifying; Seq: sequencing. ^∗^For amplification, P2_F1 forward primer was used. ^*#*^For amplification, P2_F2 forward primer was used.*

### Study 1: Identification of Differentially Methylated CpG Sites in the Canine *OXTR* Promoter

The aim of Study 1 was to identify differentially methylated CpG sites in the promoter of the *OXTR* that show a variation between individuals higher than 10%. As no prior information was available regarding localization of differentially methylated CpG sites in the canine *OXTR* gene, the DNA methylation profiles needed to be explored first. A diverse sample set including both wolves and dogs of different breeds, sex, age and keeping conditions were used during this pilot study to gain more insight into the methylation levels of the canine *OXTR* promoter region. The rationale behind choosing a heterogeneous population for this goal was that in a homogeneous sample, potential variably methylated sites are more likely to be missed, especially if the sample size is small. Given that methylation status of promoter-associated CpG islands and their immediate flanking regions, the CpG island shores, often influences gene expression and because the latter have been shown to be frequently differentially methylated (Doi et al., 2009; Portela and Esteller, 2010; Deaton and Bird, 2011), we focused on identifying differentially methylated CpGs at near promoter CpG island shore.

#### Subjects

Twelve animals (nine dogs and three timber wolves, six females and six males, mean age ± SD = 47.94 ± 37.84 months; see **Table 2** for all details about the subjects of Study 1) were involved in the present study. All wolves and two dogs were born in captivity and were hand-raised in peer-groups at the Wolf Science Center^1^ after being separated from their mothers before they were 10 days old. Among the remaining seven dogs, one lived at a Hungarian dog school as guarding dog, six were kept as pet dogs and among them four lived inside the house and two lived mainly outside.

**Table 2:** Animals involved in the identification of the differentially methylated CpG sites (Study 1).

Sub-species	Breed	Living conditions	Sex	Age
Wolf	Timber	Hand-raised at the WSC	Male	6 years
Wolf	Timber	Hand-raised at the WSC	Male	2 years
Wolf	Timber	Hand-raised at the WSC	Female	4 years
Dog	Mix breed	Hand-raised at the WSC	Female	2 years
Dog	Mix breed	Hand-raised at the WSC	Female	3 weeks
Dog	Mix breed	Pet dog (kept inside)	Female	7 years
Dog	Shetland Sheepdog	Pet dog (kept inside)	Female	2 years
Dog	Caucasian Shepherd	Guard dog at dog school	Male	7 years
Dog	Boxer	Pet dog (kept inside)	Male	6 months
Dog	Central Asian Shepherd	Pet dog (kept outside)	Male	2 weeks
Dog	West Highland White Terrier	Pet dog (kept inside)	Male	9 years
Dog	Beagle	Pet dog (kept outside)	Female	5 years


#### Data Analysis

We used the traditional CpG island definition (at least 200 bp long DNA stretch with a G+C content of at least 50% and a ratio of observed to statistically expected CpG frequencies of at least 0.6) to identify CpG islands.

#### Results

**FIGURE 1:**
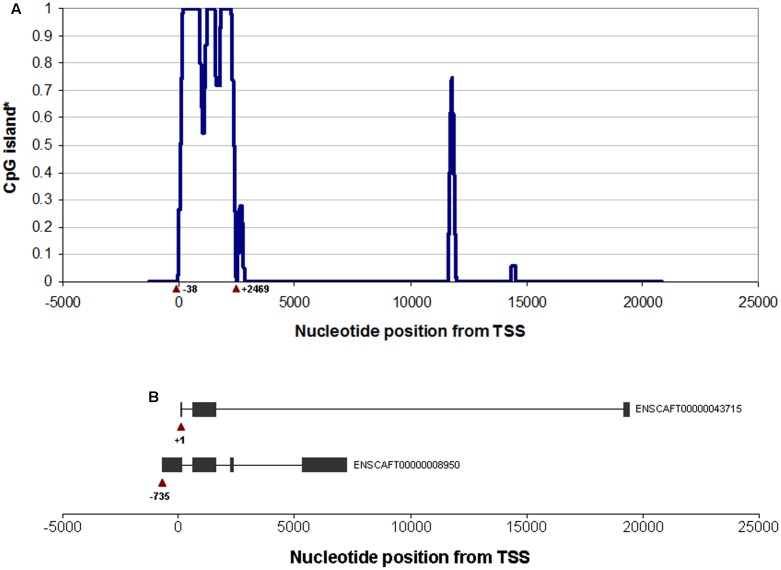
**CpG island structure of the canine OXTR gene.** **(A)** Nucleotide positions according to transcription start site of transcript variant NM_001198659.1 (ENSCAFT00000008950.3) to which the traditional CpG island definition applies (“an at least 200 bp long DNA stretch with *a* ≥ 50% G+C content and *a* ≥ 0.6 observed-to-expected CpG ratio”). ^∗^Y axis shows the proportion of all possible different 200 bp long DNA stretches containing the same nucleotide to which the described CpG island definition applies. Positions of nucleotides at the beginning and the end of the CpG island located right at the beginning of the OXTR gene are indicated with respect to +1 as the transcription start site of transcript variant NM_001198659.1 (ENSCAFT00000008950.3). **(B)** Schematic structure of the transcript variants ENSCAFT00000008950.3 (NM_001198659.1) and ENSCAFT00000043715.1. Boxes represent exons, lines represent introns. Nucleotide positions of the transcription sites are indicated relative to transcription start site of NM_001198659.1 (ENSCAFT00000008950.3).

**FIGURE 2:**
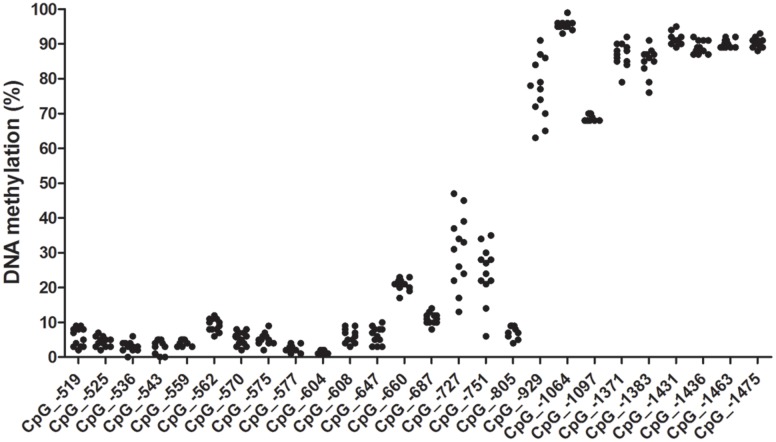
**CpG methylation levels in the canine *OXTR* promoter.** Methylation ranges indicated are as observed in the exploratory sample set of 12 animals of different subspecies, breed, sex, age and keeping conditions. CpG numbering is according to transcription start site of +1 of transcript variant NM_001198659.1. Methylation levels for CpGs –451 to –489 are not indicated due to poor sequence quality, but apparently they were all in the low methylation level range. CpG –590 was not covered by any sequencing primers.

**FIGURE 3:**
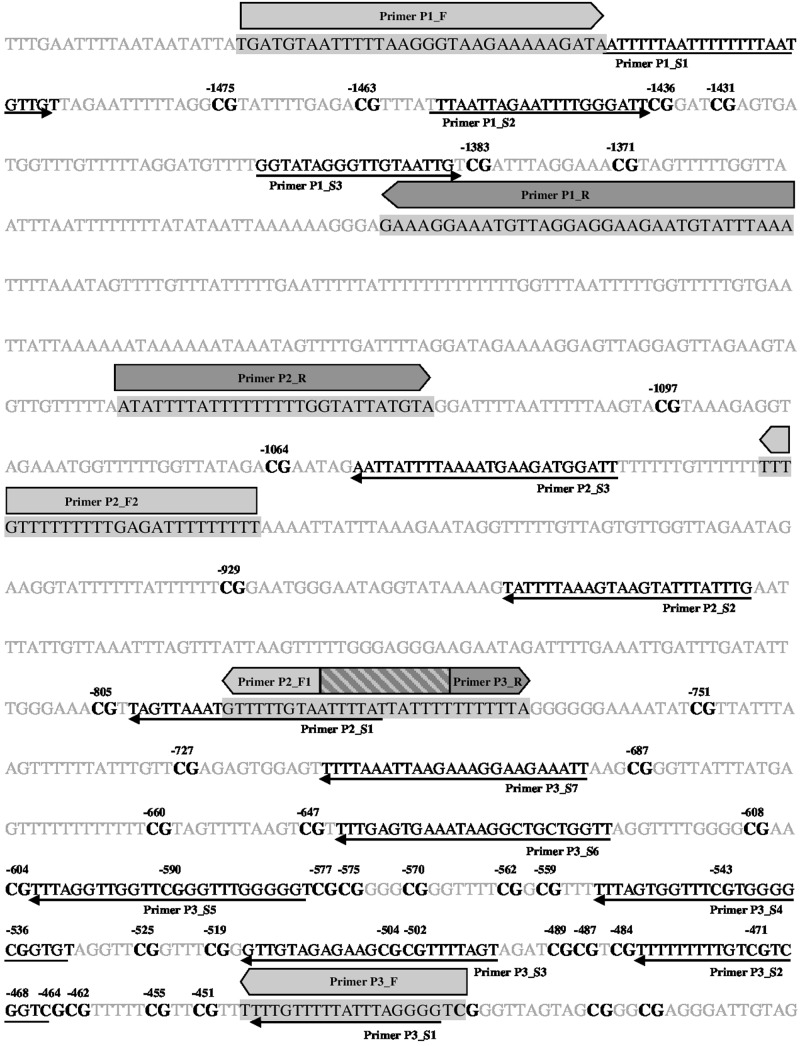
**Localization of primers and CpG sites covered.** Bisulfite converted sequence of forward strand is indicated. Primer pairs P2_F1/ P2_F2 & P2_R as well as P3_F & P3_R were designed to the reverse strand. Forward and reverse amplifying primers are indicated by filled light and darks arrows, respectively, as well as by black letters in the sequence highlighted by a gray background; sequencing primers are indicated by thin arrows as well as black italics letter in the sequence. Overlap region of forward primer P2_F1 and reverse primer P3_R is indicated by a striped box. CpG sites are shown in bold black letters. CpGs covered by sequencing primers are numbered according to transcription start site of +1 of transcript variant NM_001198659.1 [ENSCAFT00000008950.3; genomic coordinate: CanFam3.1 (GCA_000002285.2) Chr20:9358932].

We identified a CpG island located right at the 5′ UTR of the canine *OXTR* gene (**Figure 1**). Accordingly, an 1117 bp long segment located at a CpG island shore in the canine *OXTR* promoter was investigated for variably methylated CpG sites. Range of methylation levels for given CpG sites are shown in **Figure 2** (not all CpG sites were covered by pyrosequencing analysis due to difficulties of designing effective primers for bisulfite converted DNA). Out of the 26 CpG sites analyzed, four were found that showed at least 10% variation in their methylation levels among the subjects, presented with accurate methylation levels for the 0 and 100% methylated controls and their different ratio mixtures (between 10 and 90%) as well as gave high peaks in the chromatogram even in the case of low (10–12 ng) initial (pre-PCR) DNA quantities. These four CpG sites were located -727, -751, -1371, and -1383 bp relative to transcription start site of transcript variant NM_001198659.1 (ENSCAFT00000008950.3), see **Figure 3**, with their genomic coordinates being Chr20:9358205, Chr20:9358181, Chr20:9357561, and Chr20:9357549, respectively, according to genome assembly CanFam3.1 (GCA_000002285.2). These four CpG sites were then analyzed in Study 2 (see below). Genomic alignment of the canine *OXTR* promoter segment investigated to the corresponding human sequence is shown in Supplementary Figure 1.

### Study 2: Associations between Owner Interaction Style, Dog Behavior, and Methylation of the *OXTR* Promoter

#### Subjects

Study 2 originally involved 220 pure bred Border Collies but three individuals were excluded from the present analyses since it was not possible to obtain DNA samples from them. A single breed was involved to minimize background genetic variability. The 217 dogs (135 females (45 neutered) and 95 males (32 neutered); mean age ± *SD* = 48.07 ± 42.43 months) involved in the study were all kept as pets in Vienna (Austria) and surroundings. These subjects, together with their owners, participated in a behavioral test battery accompanied by buccal DNA sample collection. All subjects were tested at the Clever Dog Lab (Vienna, Austria) between September 2010 and November 2013. The owners were recruited from the database of volunteer participants of the Clever Dog Lab.

#### Behavioral Test Battery

The behavioral data were collected as part of a bigger project, and the methods and some of the results have been described by Cimarelli et al. (2016). In summary, the pet Border Collies (*N* = 217) participated in a modified version of the Threatening Approach test (Vas et al., 2005; Gácsi et al., 2013; Hernádi et al., 2015): the owner stood motionless behind the dog and held the leash. The experimenter (E), initially standing five meters away from the owner-dog dyad, started walking toward the dog slowly (approximately 1 step/4 s) with the upper body bent toward the dog and staring in the eyes of the dog. The test was over when E reached the dog, the dog approached E in a friendly manner, or when the dog showed strong signs of aggression and/or fear (i.e., snapped at E or hid behind the owner). At the end of the test, E crouched down and talked gently to the dog to resolve the situation. We analyzed whether the dog showed any of the following behaviors before E crouched down (recorded as binomial variables): friendly (approaching E wagging the tail), appeasing (approaching E with the tail between the legs, ears pulled back and tense body posture), aggressive (growling, snarling or snapping at E), passive (no visible reaction) and hiding behind the owner (withdrawing in a way that the owner would be positioned between the dogs and E). In addition, we also scored the final reaction of the dogs showed when E made the last step toward them (1 = retreat behind the owner; 2 = passive behavior; 3 = appeasing/friendly approach; 4 = aggressive approach).

Aside from analyzing the dogs’ behavior in this test, the owners’ interaction style with their dog was also characterized (Owner interaction style test). The behavior of the owner toward her/his dog was observed and coded in a set of 8 experimental tasks. The tasks included: (1) showing a preference toward one of two plates to the dog (“Food choice”); (2) holding the dog while the experimenter was taking a buccal sample from the inner mouth of the dog (“DNA sample”); (3) greeting after a short period of separation (“Greeting”); (4) playing with the dog using a rope in a tug-of-war game (“Tug-of-war”); (5) putting a T-shirt on a dog (“T-shirt”); (6) commanding the dog to perform three simple behaviors (i.e., sit, lay down, stay; “Commands”); (7) demonstrating the dog how to remove the lid from a bin to get a piece of food (“Teaching”); (8) playing a retrieval game using a ball (“Ball”). The following variables were measured: communication style (4-point scale, in Food choice and Teaching tests), warmth (4-point scale, in the Greeting test), enthusiasm (4-point scale, in the Tug-of-war and Ball test), social support (4-point scale, in DNA sample and T-shirt test), authoritarian behaviors (0 = none, 1 = the owner raises the tone of voice, 2 = the owner forces the dog in a determined position in the Commands test). Furthermore, the number of commands, attention sounds (e.g., clapping the hands), vocal praises and petting were counted in the DNA sample, Tug-of-war, Commands and Ball tests. Previous analyses showed that the behavioral variables analyzed during this test grouped in three factors, namely the “Owner Warmth” (characterized by a positive and warm communication and interaction style showed in positive contexts, e.g., play), “Owner Social Support” (characterized by the number of petting and praising given by the owner in stressful situations, e.g., DNA sample test) and “Owner Control” [mainly characterized by the number of commands; for a detailed description see (Cimarelli et al., 2016)].

#### Statistical Analysis

In order to estimate whether the methylation levels of the adjacent sites identified in Study 1 were correlated, Pearson correlations between sites were calculated. To investigate whether the Owner interaction styles and the demographic characteristics of the dog (i.e., sex, age, and neutered status) were associated with the methylation levels of the CpG sites identified in Study 1, we ran Generalized Least Squares models (GLSs) with the methylation levels as dependent variable and the Owner interaction styles and the dog demographic variables as predictors [R package nlme (Pinheiro et al., 2007), function *gls*]. Furthermore, to investigate associations between the methylation levels and dog behavior during the Threatening Approach test, we ran Generalized Linear Models (GZLM) with binomial distributions. We ran models with the methylation levels of the different CpG sites as predictors and the following variables as response variables: “Aggression,” “Appeasing,” “Friendly,” “Hide behind the owner,” and “Passive.” Furthermore, we ran a Multinomial Regression Model with the “Reaction at the end of the test” as dependent variable and the methylation levels as predictors. We selected the best model using model reduction based on *p*-values. Non-significant predictors (*p* > 0.05) were removed from the model and are not reported in the results. Model residuals were tested for normality using the Shapiro-Wilk normality test and homoscedasticity was assessed via plots of residuals against fitted values. We accounted for multiple testing using *post hoc* sequential Bonferroni (Holm, 1979). All statistical tests were conducted using R version 3.1.1 (R Development Core Team). See Supplementary Materials for a complete correlation matrix between all variables included in the present study.

#### Results

##### Characteristics of *OXTR* Promoter CpG Site Methylation

The four sites identified in Study 1 were further investigated in the Border Collie group (*N* = 217). The degree of methylation of these CpGs in the Border Collie population ranged between 9.0 and 58.7% (-727), 15.5 and 46.5% (-751), 80.5 and 89.7% (-1371), and 76.5 and 94.0% (-1383). Sites -1383 and -1371 were found to be moderately correlated (*r*_210_ = 0.23, *p* < 0.01) while sites -727 and -751 were strongly correlated (*r*_210_ = 0.69, *p* < 0.01).

##### Associations of Methylation Levels with Sex, Age, Neutered Status, Sex^∗^Neutered Status Interaction and Owner Interaction Scores

**FIGURE 4:**
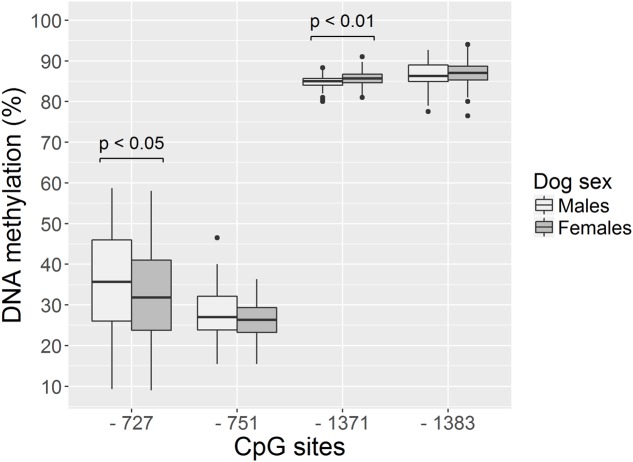
**Sex differences for methylation levels –727, –751, –1371, –1383 in dogs involved in Study 2.** Females have higher methylation levels than males at position –1371 while males have higher methylation levels than females at position –727. Horizontal bars represent medians, the bottom and the top of the boxes represent the lower and the upper quartiles, respectively, whiskers represent the interquartile range and filled circles represent outliers.

The three Owner interaction style factors, together with the dogs’ sex, age, neutered status and sex^∗^neutered status were investigated as predictors for methylation levels of the three CpG sites. We found that the none of the predictors was significantly associated with the methylation level of sites –751 and -1383 (*p* > 0.05, **Table 3** and **Figure 4**). On the other hand, the sex of the dog was associated with the methylation level in site -1367 and -723. In particular, female dogs had higher methylation levels than males in position -1371 (GLS, estimate ± *SD* = 0.81 ± 0.24, *t*_210_ = 3.31, *p* < 0.01, significant after correcting for multiple testing; **Table 3** and **Figure 4**), while males seemed to have higher methylation levels than females in position -727 (GLS, estimate ± *SD* = -3.53 ± 1.66, *t*_210_ = -2.12, *p* = 0.03; no longer significant when correcting for multiple testing; **Table 3** and **Figure 4**).

**Table 3:** Factors affecting the methylation levels of the CpG sites analyzed in Study 2.

Dependent variable	Predictor	Estimate ± *SE*	*DF*	*t* value	*p*-value	Effect size (Pearson’s *r*)
-727	Age	0.01 ± 0.02	1	0.55	0.58	0.01
	Sex	-3.53 ± 1.66	1	-2.12	0.03	0.15
	Neutered status	1.55 ± 1.87	1	0.83	0.41	0.05
	Sex^∗^Neutered status	-2.38 ± 3.86	1	-0.62	0.54	0.04
	Owner Warmth	-0.75 ± 1.16	1	-0.65	0.52	0.06
	Owner Social Support	-1.12 ± 1.10	1	-1.06	0.29	0.08
	Owner Control	-1.14 ± 1.11	1	-1.03	0.30	0.05
-751	Age	0.00 ± 0.01	1	0.43	0.67	0.03
	Sex	-1.22 ± 0.73	1	-1.67	0.09	0.12
	Neutered status	0.69 ± 0.75	1	0.92	0.36	0.06
	Sex^∗^Neutered status	-1.90 ± 1.53	1	-1.24	0.22	0.08
	Owner Warmth	0.30 ± 0.50	1	0.06	0.95	0.02
	Owner Social Support	-0.49 ± 0.47	1	-1.06	0.29	0.05
	Owner Control	0.18 ± 0.48	1	0.38	0.71	0.03
-1383	Age	0.00 ± 0.01	1	0.86	0.39	0.06
	Sex	0.37 ± 0.46	1	0.81	0.42	0.03
	Neutered status	-0.73 ± 0.49	1	-1.51	0.13	0.06
	Sex^∗^Neutered status	0.03 ± 0.95	1	0.04	0.97	0.00
	Owner Warmth	0.27 ± 0.31	1	0.88	0.38	0.07
	Owner Social Support	-0.06 ± 0.30	1	-0.20	0.84	0.02
	Owner Control	0.00 ± 0.31	1	0.01	0.99	0.04
-1371	Age	0.00 ± 0.00	1	0.03	0.97	0.08
	Sex	0.81 ± 0.24	1	3.31	0.001*	0.22
	Neutered status	-0.49 ± 0.26	1	-1.90	0.06	0.12
	Sex^∗^Neutered status	-0.34 ± 0.58	1	-0.58	0.56	0.04
	Owner Warmth	0.03 ± 0.17	1	0.16	0.87	0.07
	Owner Social Support	0.19 ± 0.16	1	1.18	0.24	0.02
	Owner Control	-0.07 ± 0.17	1	-0.42	0.67	0.04


*^∗^Significant after *post hoc* sequential Bonferroni correction for multiple testing.*

##### Associations of Methylation Levels with Dog Reaction in Males and Females

**FIGURE 5:**
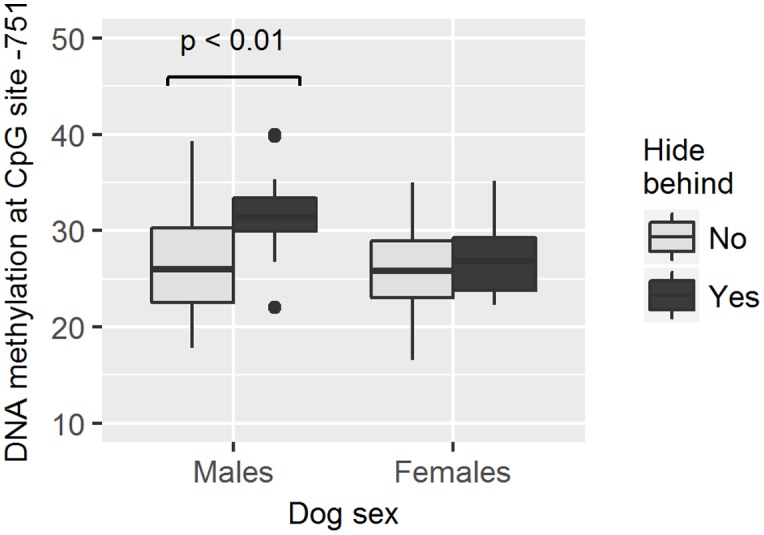
**Association between methylation levels at position –751 and the likelihood of dogs to hide behind the owner during the Threatening Approach test.** Males hiding behind the owner had higher methylation levels than males not hiding behind the owner. Horizontal bars represent medians, the bottom and the top of the boxes represent the lower and the upper quartiles, respectively, whiskers represent the interquartile range and filled circles represent outliers.

**FIGURE 6:**
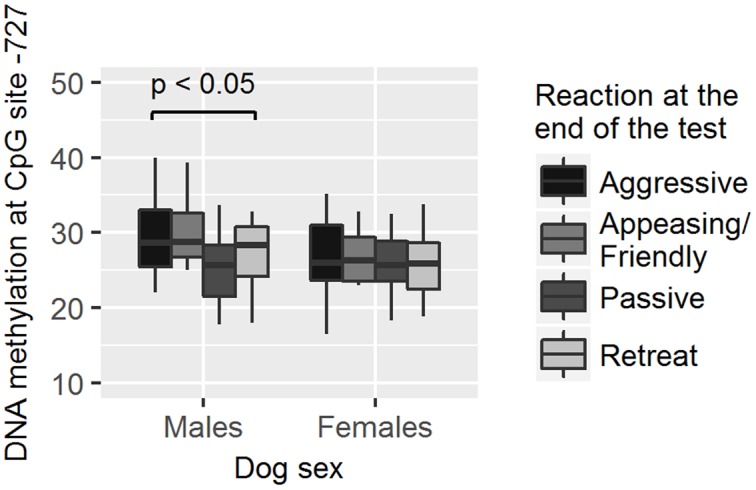
**Association between methylation levels at position –727 and their reaction at the end of the Threatening Approach test.** Male dogs approaching the experimenter either in an aggressive or in an appeasing/friendly manner had higher methylation levels than male dogs that remained passive or retreated. Horizontal bars represent medians, the bottom and the top of the boxes represent the lower and the upper quartiles, respectively, whiskers represent the interquartile range and filled circles represent outliers.

**FIGURE 7:**
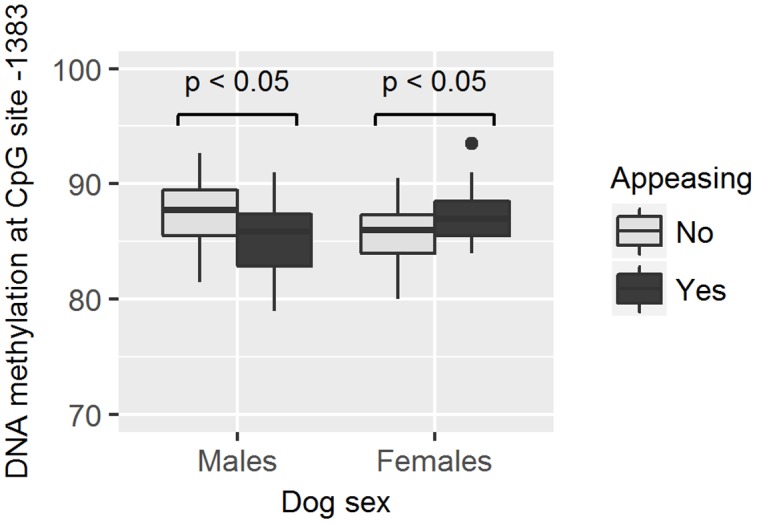
**Association between methylation levels at position –1383 and the likelihood of dogs to approach the experimenter in an appeasing way.** Males approaching the experimenter in an appeasing way had lower methylation levels than males who did not, while the opposite was true for female dogs. Horizontal bars represent medians, the bottom and the top of the boxes represent the lower and the upper quartiles, respectively, whiskers represent the interquartile range and filled circles represent outliers.

**FIGURE 8:**
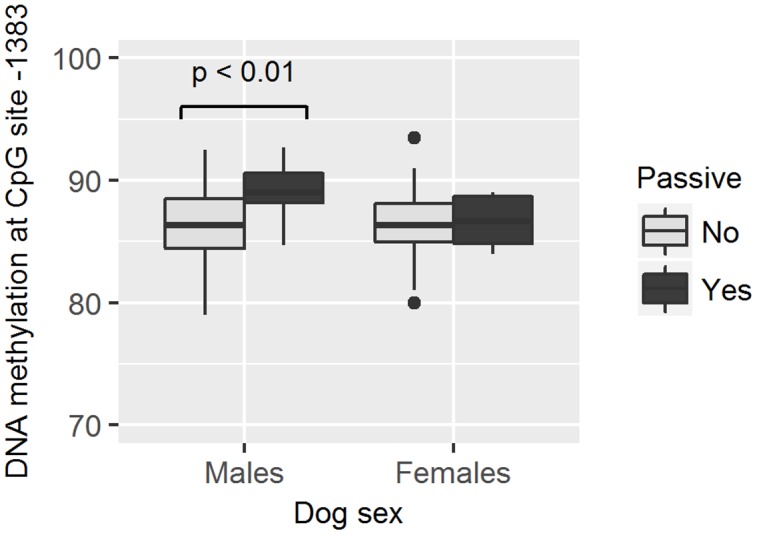
**Association between methylation levels at position –1383 and the likelihood of dogs to stay passive during the Threatening Approach test.** Males remaining passive had higher methylation levels than males showing any other reaction. Horizontal bars represent medians, the bottom and the top of the boxes represent the lower and the upper quartiles, respectively, whiskers represent the interquartile range and filled circles represent outliers.

As methylation levels were found to differ by dog sex, the association between different methylation levels and the dog behavior was analyzed separately in female and male dogs. We found that males who hid behind the owner had higher methylation levels in site -751 than those who did not hide behind the owner (GZLM, estimate ± *SD* = 0.13 ± 0.05, *z*_70_ = 2.59, *p* < 0.01, significant after correcting for multiple testing; **Table 4** and **Figure 5**) and that males remaining passive or retreating at the end of the test tended to have lower methylation levels in site -727 than males approaching the experimenter in an appeasing or aggressive manner (Multinomial Regression Model, *X*^2^ = 8.30, *df* = 3, *p* = 0.04; no longer significant when correcting for multiple testing; **Table 5** and **Figure 6**). Furthermore, females who approached the experimenter in an appeasing way tended to have higher levels of methylation in site -1383 (GZLM, estimate ± *SD* = 0.14 ± 0.07, *z*_104_ = 1.97, *p* = 0.04, no longer significant after correcting for multiple testing; **Table 4** and **Figure 7**) than those who did not show any sign of appeasement, contrary to the males who approached the experimenter in an appeasing manner which tended to have lower methylation levels in site -1383 than those who did not (GZLM, estimate ± *SD* = -0.16 ± 0.08, *z*_70_ = -2.09, *p* = 0.04, no longer significant after correcting for multiple testing; **Table 4** and **Figure 7**). On the other hand, males who remained passive till the end of the Threatening Approach test had higher methylation levels in site -1383 than those who showed any other reaction (GZLM, estimate ± *SD* = 0.43 ± 0.15, *z*_77_ = 2.81, *p* < 0.01, significant after correcting for multiple testing; **Table 4** and **Figure 8**). All non-significant associations are reported in **Tables 4**, **5**.

**Table 4:** Factors affecting male and female dogs’ reaction during the Threatening Approach test (Study 2).

Dependent variable	Predictor	Sex	Estimate *+ SE*	*DF*	*z* value	*p*-value	Effect size (Pearson’s *r*)
Aggression	-727	Males	-0.00 ± 0.05	1	-0.02	0.99	0.07
		Females	0.00 ± 0.03	1	0.05	0.96	0.07
	-751	Males	-0.05 ± 0.06	1	-0.92	0.36	0.08
		Females	-0.00 ± 0.08	1	-0.04	0.97	0.04
	-1371	Males	-0.17 ± 0.16	1	-0.01	0.31	0.13
		Females	0.21 ± 0.16	1	1.31	0.19	0.10
	-1383	Males	0.01 ± 0.11	1	0.09	0.93	0.06
		Females	-0.12 ± 0.10	1	-1.18	0.24	0.08
Friendly	-727	Males	-0.04 ± 0.06	1	-0.70	0.48	0.14
		Females	0.05 ± 0.03	1	1.50	0.13	0.15
	-751	Males	0.01 ± 0.41	1	0.03	0.98	0.10
		Females	0.05 ± 0.09	1	0.54	0.59	0.16
	-1371	Males	0.14 ± 0.46	1	0.31	0.75	0.03
		Females	0.33 ± 0.19	1	1.71	0.09	0.13
	-1383	Males	-0.11 ± 0.45	1	-0.24	0.81	0.11
		Females	-0.03 ± 0.16	1	-0.19	0.85	0.12
Appeasing	-727	Males	-0.00 ± 0.04	1	-0.07	0.94	0.14
		Females	0.02 ± 0.02	1	1.21	0.23	0.18
	-751	Males	-0.01 ± 0.04	1	-0.19	0.85	0.08
		Females	-0.06 ± 0.06	1	-1.01	0.31	0.05
	-1371	Males	0.03 ± 0.16	1	0.18	0.86	0.06
		Females	0.05 ± 0.14	1	0.39	0.69	0.02
	-1383	Males	-0.16 ± 0.08	1	-2.09	0.04	0.28
		Females	0.14 ± 0.07	1	1.97	0.05	0.22
Passive	-727	Males	-0.02 ± 0.04	1	-0.51	0.61	0.11
		Females	-0.03 ± 0.03	1	-1.09	0.27	0.04
	-751	Males	0.03 ± 0.11	1	0.30	0.76	0.09
		Females	0.07 ± 0.09	1	0.78	0.43	0.01
	-1371	Males	-0.37 ± 0.24	1	-1.55	0.12	0.04
		Females	-0.09 ± 0.21	1	-0.44	0.66	0.04
	-1383	Males	0.43 ± 0.15	1	2.81	0.005*	0.31
		Females	0.17 ± 0.11	1	1.54	0.12	0.13
Hide behind	-727	Males	-0.03 ± 0.03	1	-0.91	0.36	0.20
		Females	-0.01 ± 0.02	1	-0.33	0.74	0.04
	-751	Males	0.13 ± 0.05	1	2.59	0.009*	0.35
		Females	0.03 ± 0.07	1	0.4	0.69	0.00
	-1371	Males	-0.14 ± 0.19	1	-0.72	0.47	0.10
		Females	-0.02 ± 0.13	1	-0.16	0.87	0.05
	-1383	Males	0.03 ± 0.13	1	0.22	0.82	0.04
		Females	0.05 ± 0.10	1	0.51	0.61	0.03


*^∗^Significant after *post hoc* sequential Bonferroni correction for multiple testing.*

**Table 5:** Factors affecting dog’s reaction at the end of the Threatening Approach test (Study 2).

Dependent variable	Predictor	Sex	Level: Estimate ± *SE*	*DF*	*X*^2^	*p*-value	Effect size (Pearson’s *r*)
**Reaction at the end of the test**	-727	Males	(1) -0.01 ± 0.04	3	8.30	0.04	0.19
			(2) -0.05 ± 0.03				
			(3) -0.06 ± 0.02				
		Females	(1) 0.01 ± 0.03	3	0.81	0.85	0.06
			(2) 0.00 ± 0.02				
			(3) 0.01 ± 0.02				
	-751	Males	(1) -0.03 ± 0.08	3	7.22	0.07	0.18
			(2) -0.08 ± 0.05				
			(3) -0.13 ± 0.05				
		Females	(1) 0.00 ± 0.07	3	1.18	0.76	0.07
			(2) -0.05 ± 0.06				
			(3) -0.03 ± 0.05				
	-1371	Males	(1) -0.03 ± 0.09	3	1.99	0.57	0.10
			(2) 0.07 ± 0.15				
			(3) -0.15 ± 0.14				
		Females	(1) -0.02 ± 0.09	3	0.69	0.87	0.06
			(2) -0.02 ± 0.09				
			(3) 0.07 ± 0.09				
	-1383	Males	(1) 0.20 ± 0.18	3	3.63	0.30	0.13
			(2) -0.11 ± 0.10				
			(3) -0.02 ± 0.09				
		Females	(1) 0.12 ± 0.12	3	3.04	0.39	0.12
			(2) 0.12 ± 0.10				
			(3) -0.01 ± 0.09				


## Discussion

The present study explored for the first time the DNA methylation patterns in canids and their associations with pet dogs’ social behavior. Specifically, four CpG sites in the *OXTR* promoter were identified where at least 10% of inter-individual variation in their methylation level was observed. The methylation levels of these CpG sites were different in female and male dogs and were associated with the behavioral reaction dogs showed when exposed to social stress. These results provide the first evidence of an association between epigenetic modifications of *OXTR* and dog social behavior. In particular, we found higher methylation levels in females than in males at site -1383 and we found a tendency to have lower methylation level at site -727 in females than in males. Moreover, lower methylation levels in this position tended to be associated with a higher likelihood to approach a threatening unfamiliar person (either in an aggressive, appeasing or friendly manner) in males and a lower likelihood to remain passive or hide behind the owner. Regarding the two sites -751 and -1383, males with higher methylation levels were more likely to remain passive or to hide behind the owner than those having lower methylation levels.

It is not surprising that we found different methylation patterns in female and male dogs. Oxytocin is a hormone also with sex-related functions, therefore its receptors are expressed differently in males and females (Alves et al., 2015). It has also been shown that oxytocin administration and oxytocin level influence the social behavior of prairie voles in a sex-specific way (Bales and Carter, 2003; Bales et al., 2007). Similarly, in our study, a tendency for a different association was found in male and female dogs in regards to the appeasing behavior; while females were more likely to approach the experimenter in an appeasing manner if their methylation levels were higher in site -1383, in males we found the opposite relationship. These results might be explained by a differential interplay between the methylation of the *OXTR* gene and other biological mechanisms (e.g., the expression of sex hormones) and/or reflect a sex-specific response strategy to social threat.

Furthermore, our results suggest that different CpG sites might be differently involved in behavioral regulation. Higher promoter methylation levels generally lead to lower *OXTR* gene expression, which, in turn, leads to fewer available receptors for the oxytocin to bind. The present study suggests that the different sites might regulate *OXTR* expression in different manners: for instance, CpG sites -727 might be located in a transcription inhibitory region, where suppression of inhibition by methylation would potentially lead to higher gene expression (Portela and Esteller, 2010), or methylation of this site could trigger the use of an alternate, potentially more active, promoter (Maunakea et al., 2010). In fact, higher methylation levels on -751 or on -1383 were associated with more owner-directed behaviors or a passive state while higher methylation levels on -727 tended to lead to the opposite behavioral outcome.

Naturally, the identified associations can only be genuine if the analyzed biomarker – *OXTR* promoter methylation at the investigated CpG sites in canine buccal epithelia – reliably refers to neural processes, regulating the *OXTR* gene expression in the brain. A direct experimental verification of such a biological connection is unfortunately highly challenging, mainly because of the limited accessibility of brain tissues of (pet) dogs. Still, indirect evidence suggests that *OXTR* promoter methylation levels as measured in buccal epithelium could indeed be of physiological relevance for behavior. Human-related studies identified strong correlation between brain and surrogate tissue DNA methylation levels regarding functionally important *OXTR* promoter CpG sites (Gregory et al., 2009; Jack et al., 2012; Bell et al., 2015; Chagnon et al., 2015; Puglia et al., 2015). As buccal epithelium is of the same germ layer origin as neural tissues (Tam and Behringer, 1997), it is plausible that the inherited component of DNA methylation states remains relatively similar during embryonic development, when basic DNA methylation patterns are established (Reik and Walter, 2001). In later life, these patterns are modified both by environmental factors and stochastic effects (Kaminsky et al., 2009; Aguilera et al., 2010; Choi and Friso, 2010). How different tissues could react to environmental stimuli in similar manners in terms of DNA methylation is yet to be elucidated. However, it has been reported in humans that, even in the case of white blood cells, dynamic changes in *OXTR* promoter methylation can be observed in response to social stimuli (Unternaehrer et al., 2012). Given that *OXTR* protein is also expressed in squamous epithelial cells according to The Human Protein Atlas (Uhlen et al., 2010, 2015), it is feasible that nerves innervating the oral epithelium directly mediate epigenetic communication between brain and buccal tissues (Kress et al., 2006; Kim et al., 2009; da Silva et al., 2015). It is important to mention, however, that it has been shown that *OXTR* promoter methylation in rodents brain affects transcription efficiency in a region-specific manner (Harony-Nicolas et al., 2014). Future studies should investigate associations between *OXTR* methylation in brain tissues and in buccal cells and tissue-specific oxytocin expression in order to fully inform the psychophysiological role of *OXTR* methylation in the buccal epithelium in dogs.

Contrary to our predictions, in the present study, we could not find any association between owner behavior and methylation levels of the *OXTR* gene of their dogs. It might be that the methylation profiles of the CpG sites investigated in the present study are mostly inherited (Reik and Walter, 2001) and/or not be representative of the methylation levels of the *OXTR* gene in brain tissues that could still be potentially affected by the environment. In addition, it is possible that the owner interaction styles analyzed in the present study were factors not strong enough for such methylation changes. Indeed, the present analyses were carried out in a rather uniform population of purebred Border Collies kept as pets, and it would be necessary to investigate different breeds and/or dogs living in different social environments (e.g., in shelters or as stray dogs) in order to further investigate the role of the environment in shaping dog social behavior through epigenetic modifications. Further on, in this study we focused on the promoter of the *OXTR* gene, and we cannot exclude the possibility that owners’ interaction styles might affect other regulatory regions in other genes.

It is important to notice that the *OXTR* methylation might not be the only factor to influence dog behavior, it is possibly also mediated by which SNPs the dog was carrying (Smearman et al., 2016). In fact, some studies highlighted a correlation between degree of methylation and SNPs (Bell et al., 2012; Smith et al., 2015). In our study, we did not take into account the genetic background of our subjects, but future studies should address the interaction between environment, SNPs, DNA methylation and behavior in order to have a better understanding of the mechanisms regulating dog social behavior. Epigenetic modifications other than DNA methylation should also be investigated. It is also important to note that the pyrosequencing technique used for DNA methylation assessment is not suitable for differentiating between 5-methylcytosines and 5-hydroxymethylcytosines (Guibert and Weber, 2013), so it cannot be ascertained yet if (some of) the observed relationships are not linked to hydroxymethylation. Another important issue is that even when considering only a single epigenetic mark (DNA methylation) and a single gene, it would be useful to obtain data regarding the whole gene and all of its regulatory regions, i.e., not only a limited number of CpG sites in the promoter region. The present tissue choice (buccal epithelia) is unfortunately not suitable for such a comprehensive analysis mainly because of the obtainable DNA yield. Though buccal tissue has the major advantage of offering non-invasive sample taking and thus easy accessibility and keeping physiological effects of the sample taking procedure itself to a minimum, future studies should consider the use of other tissues as well in order to ensure investigation of a larger number of CpG sites within the same population.

Social behavior is a complex and multi factorial phenotype regulated by various interacting mechanisms: genetic background as well as inherited and environmentally induced epigenetic modifications of the individuals. The present study focused on only one of the possible mechanisms, namely the methylation of a single gene promoter, without clearly disentangling between inherited or environmentally influenced epigenetic patterns. As such, our results can provide an initial contribution to shedding light on the complex processes shaping social behavior. In particular, by indicating epigenetic analyses as a novel tool for the understanding of the mechanisms regulating dog behavior and ultimately suggesting pet dogs as good models for the field of human epigenetics. Future studies would need to investigate the interactions between the methylation levels and the polymorphisms of *OXTR*, the correspondence between buccal DNA methylation states of the CpG analyzed here and those in different regions of the brain, the effect of methylation in those areas on nervous system functions and on dog behavior, and other environmental factors possibly influencing epigenetic modifications.

## Author Contributions

GC, ZB, ZV, ZR, and MS-S designed the study. GC, ZB, BT, ZV, ZR, and MS-S prepared the study material and data acquisition. GC, ZB, and BT entered the data and prepared it for statistical analyses. GC, ZB, and BT analyzed the data. GC, ZB, BT, and ZV interpreted the data. ZV, ZR, and MS-S obtained funding. GC and ZB wrote the first draft of the manuscript. GC, BT, ZB, and ZV critically revised the manuscript for important intellectual content. All authors gave final approval of the manuscript version to be published and agreed to be accountable for all aspects of the work in ensuring that questions related to the accuracy or integrity of any part of the work are appropriately investigated and resolved.

## Conflict of Interest Statement

The authors declare that the research was conducted in the absence of any commercial or financial relationships that could be construed as a potential conflict of interest.

## Footnotes

1 www.wolfscience.at
