# Supplementary material for: Social Behavior of Pet Dogs Is Associated with Peripheral OXTR Methylation
Source: Front Psychol. 2017 Apr 10;8:549. doi: 10.3389/fpsyg.2017.00549 (PMC5385375; doi:10.3389/fpsyg.2017.00549)
Supplement: Supplementary file 2 [file Image_1.PDF]

Supplementary Figure 1. Genomic sequence alignment between human and dog for the regions investigated in the present study.

H_sap: Homo sapiens, C_fam: Canis familiaris.

Nucleotide numbering is according to transcription start sites (+1) of human and canine *OXTR* transcript variants NM_000916.3 (ENST00000316793.7) (genome assembly: GRCh38.p7 (GCA_000001405.22)) and NM_001198659.1 (ENSCAFT00000008950.3) (genome assembly CanFam 3.1 (GCA_000002285.2)), respectively.

CpG sites investigated in the Border Collie population are highlighted with dark grey. No polymorphisms affecting either the canine CpG sites investigated or their corresponding human sequences are indicated in public databases.

The presence of an asterisk (*) indicates nucleotide match between the corresponding human and canine sequences.

```
OXTR_H_sap T----TGACT---AATATT--GATTAATACTGCCTGCCACCCCTTGGCAA -839
OXTR_C_fam TGATGCAACTCCCAAGGGCAAGAAAAAGACAACTT-CCAACTCTTCCCAA -1495
           *      ***   **      **  ** **  * * *** * ***  ***

OXTR_H_sap TGCTGTCAAGATTCCCAGCCCCATTCTGGAATGATTACTCAGCTAGAACC -789
OXTR_C_fam TGTTGTCAGAATTCCCAGGCGTACCCTGAGACGTTCACTCAACTAGAACC -1445
           ** *****  ******** *  *  ***  * * * ***** ********

OXTR_H_sap CTGGGATCCAGGTGCTGTAA-GGTTGGCCCCTGGGATATCTCGGCATGGG -740
OXTR_C_fam CTGGGACCCGGACCGAGTGATGGTCTGTCCCTAGGATGTCCTGGCACAGG -1395
           ****** ** *     ** * ***  * **** **** **  ****  **

OXTR_H_sap GCTGTAATTGTGGATTAAGGAAACCCAGTCCTTGGCTAACTCAAGTCTCT -690
OXTR_C_fam GCTGTAATTGTCGACTCAGGAAACGCAGTCCTTGGCTAACTCAATTCTCT -1345
           *********** ** * ******* ******************* *****

OXTR_H_sap CCACATAATAAAAAGACGGAGAAGTGAAATGTCAGGAGGAAATACACATT -640
OXTR_C_fam CCACATAATTAAAAAAGGGAGAAAGGAAATGTCAGGAGGAAGAATGCATT -1295
```

```
           ********* **** * ******  ****************  *  ****

OXTR_H_sap TAATGCATTTTAAAGAGCCCTGTTTATTTTTGAATCCTGGCCTTTTTTT- -591
OXTR_C_fam TAA---ATTTTAAACAGCCTTGTTCATTTTTGAATCCTCACTTTTTTTTT -1248
           ***   ******** **** **** *************  * *******

OXTR_H_sap -CTGACTTAATTCTTGGCCACTGTAAATTACTT-----CAAAAAAT---- -551
OXTR_C_fam TCTGGTTTAATTCTTGGCCTCTGTGAATCACTAAAAAAATAAAAAATAAAT -1198
            ***  ************* **** *** ***       *******

OXTR_H_sap ------GATTTTAGAATAGAGAAGGGGCAGGGAGGCTGAGA-AGCTGTCT -508
OXTR_C_fam AGCCCTGATTTTAGGATAGAAAAGGAGTTAGGAGTCAGAAGCAGCTGTCT -1148
                 ******** ***** **** *   **** * **   ********

OXTR_H_sap TTAACATTTTATCTTCCTTTGGCATCATTTAGAATTTTAATTCCGAAGCG -458
OXTR_C_fam TTAACATTTCATCTTCCTTTGGCATCATGTAGGATTTTAATTTCCAAGCA -1098
           ********* ****************** *** ********* * ****

OXTR_H_sap CGACAAGGAGGCAGAAACGGCTCTTGGGCGCAGACAAGCAGAATCACTTT -408
OXTR_C_fam CG-CAAAGAGGCAGAAATGGCTTCTGGCCACAGACGAACAGAATCACTTT -1049
           ** *** ********** ****  *** * ***** * ************

OXTR_H_sap AAA-TGAAGACAG-----TGTTGTGCTTC--------------AGAATTT -378
OXTR_C_fam AAAATGAAGATGGATTTTTTTTGTTCTTTCTTGTCTTTTCTTGAGATCTT -999
           *** ******  *     * **** ***               ***  **

OXTR_H_sap CCTCTAAAACTACCGAAAAAATAACGCCTCTCCCAGCACTGCTTAGAATA -328
OXTR_C_fam TCCCTAAAACTACCTAAAGAATAG-GCCTCTGCCAGTGCTGGTTAGAACA -950
            * *********** *** ****  ****** ****  *** ****** *
```

```
OXTR_H_sap GAGGCCATTTCTAATTCCTCATTAACGGGAATAGGAACAAAAGTATTCCA -278
OXTR_C_fam GAAGGCATTTCTCATCCCTCCGGAATGGGAATAGGCACAAAAGTACTCCA -900
           ** * ******* ** ****   ** ********* ********* ****

OXTR_H_sap AAGCAAAGACTTATTTGAGTTCACTGCTAAAGCCGCTACATCAAGCTGGA -228
OXTR_C_fam AAGCAAGCATTTACTTGAATTCACTGCTAAATCCAGTTTATCAAGCT--- -853
           ******  * *** **** ************ **  *  ********

OXTR_H_sap GGTGTGGGGGGAGAGAAAAGCCTGAAAATTAACATCATTTTTGGGAAATA -178
OXTR_C_fam CCTGGGAGGGAAGAATAGACTCTGAAA-TTGACCTGATATTTGGGAAACG -804
             ** * *** ***  * *  ****** ** ** * ** *********

OXTR_H_sap ATCAGTTTAAATGCTTTTGTAACTTCATCACTATCTACCC--GGGGAAGA -130
OXTR_C_fam -TCAGTT-AAATGCTTTTGCAACTTCATCACCCTCTTCTCAGGGGGGAAA -756
            ****** *********** ***********  *** * *  **** * *

OXTR_H_sap ACATTATTATTCAAGCCTCCTATGTGTCTCGGAGTCAAGAGCTTCTAAAC -80
OXTR_C_fam ACACCGCTATTCAAGCCTCCCACTTGCCCGAGAGT--GGAGCTTCTAAAC -708
           ***    ************* *  ** *   ****   ************

OXTR_H_sap CAAGAAAGGAAGAAAC-GGGCGGGTTATTGACGAGTTCCCTCCCTCTCGC -31
OXTR_C_fam CAAGAAAGGAAGAAACCAAGCGGGTTATTTATGAGCTCCTTCCCTCTCGC -658
           ****************   ********** * *** *** **********

OXTR_H_sap AGTTTTAAACCACT----GCAAAATAAACCCATTTGTTAAGGCTCTGGGA +16
OXTR_C_fam AGCTTCAAGCCGCCTTGAGTGAAATAAGGCTGCTGGTTA-GGCTCTGGGG -609
           ** ** ** ** *     *  ******  *   * **** *********

OXTR_H_sap CCAACGCTGGGCGAACCAGCTCCGCTCCGGAGGGGTCTGCGCGGCTGGCC +66
OXTR_C_fam CGAACGTC---CAGGCTGGCCCGGGTCTGG--GGGTC-GCGGGGCGGGCC -565
```

```
           * ****     *   *  ** * * ** **  ***** *** *** ****

OXTR_H_sap TCGCCCGCCCCCTAGCGGACCCGTGCGATAGTGCAGCCTCAGCCCCAG-- +114
OXTR_C_fam CTCGGCGCCTCCCAGTGGCCCCGTGGGGCGGTGCAGGCCCGGCCCCGGGC -515
                **** ** ** ** ****** *   ****** * * ***** *

OXTR_H_sap CGCACAGCGCCGCATCC----AGAC---GCTGTCCGCGCGCGCAGCCTGG +157
OXTR_C_fam TGCAGAGAAGCGCGCCCCAGCAGACCGCGCCGCTTTTTTTTGCCGCC--G -467
            *** **   ***  **    ****   ** *         ** ***  *

OXTR_H_sap GAGGCGCTCCTCGCTCGCCTCCTGTACCCATCCAGCGACC +197
OXTR_C_fam GCCGCGCTCCCCGCTCGCCTCCTGTCCCCATCCAGGGGCC -427
           *  ******* ************** ********* * **
```
